# Supplementary material for: Modeling glioblastoma heterogeneity as a dynamic network of cell states
Source: Mol Syst Biol. 2021 Sep 16;17(9):e10105. doi: 10.15252/msb.202010105 (PMC8444284; doi:10.15252/msb.202010105)
Supplement: Supplementary file 5 — Source Data for Figure 3 [file MSB-17-e10105-s001.zip › Figure3A_sourcedata/GSEA_3065/hallmarks_state2.GseaPreranked.1623416269049/HALLMARK_HEDGEHOG_SIGNALING.html]

Details for gene set HALLMARK\_HEDGEHOG\_SIGNALING[GSEA]

|  || Dataset | state2 |
| Phenotype | NoPhenotypeAvailable |
| Upregulated in class | na\_neg |
| GeneSet | HALLMARK\_HEDGEHOG\_SIGNALING |
| Enrichment Score (ES) | -0.7337522 |
| Normalized Enrichment Score (NES) | -2.0808558 |
| Nominal p-value | 0.0 |
| FDR q-value | 0.0 |
| FWER p-Value | 0.0 |
Table: GSEA Results Summary

  

Fig 1: Enrichment plot: HALLMARK\_HEDGEHOG\_SIGNALING      
 Profile of the Running ES Score & Positions of GeneSet Members on the Rank Ordered List

  

| PROBE | GENE SYMBOL | GENE\_TITLE | RANK IN GENE LIST | RANK METRIC SCORE | RUNNING ES | CORE ENRICHMENT || 1 | CDK6 |  |  | 2124 | 0.060 | -0.2032 | No |
| 2 | CRMP1 |  |  | 3968 | 0.016 | -0.3930 | No |
| 3 | AMOT |  |  | 4413 | 0.009 | -0.4369 | No |
| 4 | VEGFA |  |  | 4721 | 0.004 | -0.4681 | No |
| 5 | ETS2 |  |  | 4918 | 0.001 | -0.4886 | No |
| 6 | TLE1 |  |  | 6017 | -0.017 | -0.5989 | No |
| 7 | RTN1 |  |  | 6561 | -0.026 | -0.6468 | No |
| 8 | RASA1 |  |  | 6745 | -0.030 | -0.6549 | No |
| 9 | LDB1 |  |  | 6957 | -0.035 | -0.6643 | No |
| 10 | PTCH1 |  |  | 7258 | -0.044 | -0.6799 | No |
| 11 | PML |  |  | 7505 | -0.051 | -0.6873 | No |
| 12 | TLE3 |  |  | 7678 | -0.056 | -0.6849 | No |
| 13 | THY1 |  |  | 8139 | -0.074 | -0.7064 | Yes |
| 14 | VLDLR |  |  | 8186 | -0.077 | -0.6829 | Yes |
| 15 | DPYSL2 |  |  | 8507 | -0.096 | -0.6811 | Yes |
| 16 | HEY1 |  |  | 8555 | -0.100 | -0.6490 | Yes |
| 17 | NF1 |  |  | 8661 | -0.108 | -0.6202 | Yes |
| 18 | MYH9 |  |  | 8778 | -0.119 | -0.5883 | Yes |
| 19 | OPHN1 |  |  | 8872 | -0.133 | -0.5490 | Yes |
| 20 | ADGRG1 |  |  | 9275 | -0.222 | -0.5093 | Yes |
| 21 | NRCAM |  |  | 9339 | -0.260 | -0.4196 | Yes |
| 22 | NRP2 |  |  | 9357 | -0.281 | -0.3171 | Yes |
| 23 | SCG2 |  |  | 9440 | -0.879 | 0.0000 | Yes |
Table: GSEA details [plain text format]

  

Fig 2: HALLMARK\_HEDGEHOG\_SIGNALING: Random ES distribution      
 Gene set null distribution of ES for **HALLMARK\_HEDGEHOG\_SIGNALING**
